# Pain, decisions, and actions: a motivational perspective

**DOI:** 10.3389/fnins.2013.00046

**Published:** 2013-04-02

**Authors:** Katja Wiech, Irene Tracey

**Affiliations:** Nuffield Division of Anaesthetics, Nuffield Department of Clinical Neurosciences, Centre for Functional Magnetic Resonance Imaging of the Brain, John Radcliffe Hospital, University of OxfordOxford, UK

**Keywords:** pain, modulation, goals, learning, motivation, analgesia, cognitive, affective

## Abstract

Because pain signals potential harm to the organism, it immediately attracts attention and motivates decisions and action. However, pain is also subject to motivations—an aspect that has led to considerable changes in our understanding of (chronic) pain over the recent years. The relationship between pain and motivational states is therefore clearly bidirectional. This review provides an overview on behavioral and neuroimaging studies investigating motivational aspects of pain. We highlight recent insights into the modulation of pain through fear and social factors, summarize findings on the role of pain in fear conditioning, avoidance learning and goal conflicts and discuss evidence on pain-related cognitive interference and motivational aspects of pain relief.

## Introduction

Of the various consequences our actions can have, pain is probably the strongest indicator that our behavior needs readjustment. Joint pain after a first running session, for instance, indicates that we might have to slow down, start with a shorter distance or improve our running style. Pain therefore motivates decisions and actions to prevent further harm to the organism. Its imperative character has made pain a popular tool in studies investigating different aspects of learning. The vast literature on classical and operant conditioning is difficult to imagine without noxious stimuli driving the acquisition and shaping of new behavior. More recently, noxious stimuli have also been employed in studies on other basic psychological processes such as value representation and decision-making in which pain features as an opponent to reward stimuli.

However, action implications of pain have also become the focus of research on pain itself. Pain commonly triggers withdrawal behavior that might be adaptive in acute situations but can be maladaptive if it becomes excessive. Persistent avoidance behavior in which patients, for instance try to prevent or alleviate pain by reducing physical activity, is associated with long-term negative affective outcome and, ironically, often leads to more pain. Behavioral consequences of pain (including non-overt cognitive and affective behavior) can therefore directly contribute to the maintenance of chronic pain. In contrast to research in which pain is used as a tool to investigate general principles of learning or decision-making, these investigations aim at characterizing pain-related decision and actions with a focus on their repercussions for the perception of (clinical) pain.

Last but not least, pain not only motivates behavior but is also subject to and influenced by motivational states. The same joint pain we experienced during our first running session might be negligible if it occurred while we try to escape from an assailant. The relationship between pain and motivations is therefore considered bidirectional. Over recent years, interest in the modulation of pain through cognitive and affective processes has intensified considerably and constitutes a third strand of research—this time, however, with a focus on sensory processing. Due to studies in this and related fields, pain is no longer seen as a direct reflection of incoming nociceptive information but is understood to vary depending on cognitive-affective influences, including current and long-term motivations of the individual.

For all three lines of research, behavioral studies have extensively characterized the psychological processes involved and neuroimaging studies have begun to elucidate their underlying neural basis. In most cases, these studies were able to describe neural correlates and identify brain regions that are pivotal to the respective process. However, more research is needed to depart from this rather descriptive approach and understand the neural mechanisms underlying the interaction between pain, decisions, and actions.

In this article, we will give an overview on the existing behavioral and neuroimaging literature on this interaction, introduce key theoretical concepts and models, portrait new emerging lines of research and highlight open questions that warrant further attention. In particular, we will discuss findings from neuroimaging studies investigating (1) the role of pain in fear conditioning, (2) avoidance behavior in the context of pain, (3) pain-related goal conflicts, (4) the interruptive function of pain on cognitive processes, and (5) the influence of motivational states on the perception of pain.

## Influence of pain on decisions and actions

### Pain as a primary reinforcer in associative learning

Studies investigating learning (and particularly associative learning during fear conditioning) have widely capitalized on the fact that pain motivates behavior. In fear conditioning, an individual is exposed to an initially neutral stimulus (e.g., geometric shape; conditioned stimulus, CS) that is paired with an aversive stimulus (e.g., noxious heat; unconditioned stimulus, US). As the individual learns that the CS predicts the US, the CS acquires aversive properties and is able to elicit conditioned fear responses.

Studies using formalized computational models such as the Rescorla–Wagner model or temporal difference learning have begun to elucidate the mechanisms that underlie learning. Based on numerous observations, these models assume that learning is primarily driven by the informational value of the unconditioned stimulus (US), i.e., it is enhanced when the CS is paired with an unexpected as opposed to an expected US. Critically, a discrepancy between the expected and the experienced US generates a “prediction error signal” in the brain that triggers updating of expectations (for an overview see McNally et al., 2011). Using functional magnetic resonance imaging (fMRI), Ploghaus et al. (2000) provided first evidence for both positive and negative prediction error signals in pain-related learning. Unexpected pain led to increased activity in the hippocampus, superior frontal and superior parietal lobe as well as in the cerebellum. The unexpected omission of pain, in contrast, increased the signal level in these regions except for the superior frontal lobe that showed reduced activity.

Temporal difference learning in the context of pain has been shown by Seymour and colleagues. In a second-order cue learning task, participant were presented with two consecutive visual cues that predicted the application of a high or low-intensity noxious stimulus (Seymour et al., 2004). On some of the trials, the expectation that had been induced by the first cue was revised by the second cue that was fully predictive in all trials. Prediction error processing following cue update was reflected in increased activation in the anterior insula and the ventral striatum. In a second study using a classical conditioning paradigm in healthy volunteers in which visual cues predicted the termination of tonic pain, Seymour et al. (2005) showed that learning about pain relief follows reward-like learning signals found in the amygdala and midbrain. The exacerbation of pain, in contrast, could be described by aversion-like signals in the orbitofrontal and anterior cingulate cortices. In a recent study, the same authors investigated prediction error processing in a decision-making task (Seymour et al., 2012). On each trial, participants had to choose one of four options, which were associated with different probabilities to receive monetary reward or a noxious stimulus. Pain-related prediction error processing was negatively correlated with activation of the striatum while the reward-related prediction error showed a positive correlation with activation in the same region.

Experimental studies on fear conditioning commonly use exteroceptive stimuli such as visual or auditory stimuli as the CS. These stimuli are deliberately chosen to be abstract and neutral (e.g., abstract shapes or white noise) as they are intended to only become meaningful (i.e., predictive) through the association with the US. In many clinical conditions including anxiety disorders and chronic pain, however, symptoms are more commonly predicted by natural interoceptive and proprioceptive stimuli. Interoceptive stimuli provide afferent information from receptors that monitor the internal state of the body, e.g., migraine aura, stiff joints, or a general feeling of discomfort. Interoceptive fear conditioning therefore occurs when an association between an interoceptive CS and a US (e.g., pain) has been established (De Peuter et al., 2011). Despite its clinical relevance, interoceptive conditioning and its role in the development and maintenance of chronic pain has only received very little attention so far.

First studies, have, however, begun to explore the influence of proprioception that is defined as the perception of posture and movement. Proprioceptive fear conditioning is particularly relevant in patients with pain in the musculoskeletal system. Fear of movement, for instance, is a strong predictor of self-reported disability (Crombez et al., 1999). In a recent study, Meulders et al. (2011) demonstrated the acquisition of fear of movement-related pain through associative learning in healthy subjects. In a fear conditioning paradigm, a particular joystick movement served as a conditioned stimulus (CS) that was followed by a painful electrical stimulus (CS+). A second movement was not associated with the noxious stimulation (CS−). Over time, the CS+ movement started to elicit a conditioned fear response, as indicated by fear-potentiated eyeblink startle responses and increased fear of pain ratings following the CS+ movement. Longer response latencies for CS+ movements suggest that as a consequence participants became more reluctant to initiate the CS+ movement or were inclined to avoid the CS+ movement.

In a first attempt to investigate neural responses induced by proprioceptive cues, Barke et al. (2012) presented chronic low back pain (CLBP) patients and healthy controls with pictures showing back-straining or neutral movements. As expected, the patient group rated the back-straining pictures as more negative and arousing. However, brain responses acquired with fMRI did not reveal any group differences in the interaction analysis. Holtz et al. (2012) used fMRI to investigate the anticipation of a hyperventilation task as an interoceptive threat. When healthy subjects were presented with a visual cue that signaled the hyperventilation task, increased activation was found in the anterior insula, orbitofrontal cortex (OFC) and mid cingulate cortex (MCC), resembling findings on the anticipation of exteroceptive stimuli (e.g., Wiech et al., 2010).

Despite its long-standing history, research on associative learning and its relevance for chronic pain will remain a topic of interest with many facets. In addition to learning about interoceptive and proprioceptive cues discussed above, associated research lines have, for instance, begun to explore the generalization of fear responses to stimuli that resemble the CS (Lissek, 2012) or aim at understanding extinction learning to improve therapeutic interventions targeting learned maladaptive responses (Milad and Quirk, 2012).

### Pain and avoidance learning

Learning about cues that predict pain enables us to avoid pain before it occurs. The clinical syndrome termed asymbolia that is characterized by a blunted reaction to pain and the lacking motivation to avoid or reduce pain exemplifies the biological significance of this motivational component. Patients with pain asymbolia commonly present with severe injuries that not only relate to the initial trauma but also to the lack of subsequent protective behavior as the physical harm does not trigger actions that are required to restore physical integrity. Although avoidance behavior might be beneficial in acute situations, it can be detrimental if it becomes excessive. For chronic pain patients, excessive avoidance behavior has been shown to exacerbate pain (see Vlaeyen and Linton, 2000; Leeuw et al., 2006 for review) and the degree of avoidance behavior is a strong predictor of pain-related disability (Karsdorp and Vlaeyen, 2009).

According to psychological models, the maintenance of avoidance behavior can mainly be explained by its ability to reduce fear. Because pain-predictive cues trigger fear and anxiety, avoiding these cues promises the escape from these negative emotional states. The aim of avoidance strategies is therefore not only to prevent pain but to avoid the aversive anticipatory state associated with it. The dual process theory (Mowrer and Lamoreaux, 1946) therefore posits that avoidance learning comprises two stages: the initial phase in which we learn about predictive cues through associative learning and the second phase in which avoidance behavior is reinforced and maintained by fear reduction following the principles of operant conditioning. Critically, avoidance behavior minimizes the opportunities to learn that the feared stimulus or event is no longer associated with pain—an implication that makes avoidance behavior particularly resistant to extinction. A key intervention in cognitive behavioral therapy (CBT) approaches to avoidance behavior is therefore the exposure to feared stimuli or events to break the vicious circle of avoidance and symptom maintenance.

Experimental studies approach avoidance learning by investigating responses to cues that predict the omission or absence of adverse outcome. Neuroimaging studies using this paradigm have shown that avoidance learning critically involves the amygdala (Schlund and Cataldo, 2010; Prévost et al., 2011). The presentation of cues that signaled the possibility to avoid future money loss or escape from immediate escalating money loss both led to increased activation of this structure (Schlund and Cataldo, 2010). Although additional brain regions such as the striatum and hippocampus have been implicated in avoidance learning (Schlund et al., 2011), their role is considerably more controversial.

Intriguingly, the neural circuitry underlying avoidance learning substantially overlaps with the one underlying approach learning. Visual cues that signal trials of potential monetary gain and those signaling avoidance of monetary loss both induced increased activation in prefrontal regions, insula, anterior cingulate cortex (ACC), amygdala, hippocampus, and parahippocampus (Schlund et al., 2011). This strong resemblance of activation patterns has led to the hypothesis that similarly to positive outcome, avoidance might be rewarding. Support for this notion comes from studies investigating brain responses during the presentation of choice outcome. Delivery of monetary reward and the omission of monetary loss were associated with comparable activations in frontal and striatal regions (Schlund et al., 2011). In a study by Kim et al. (2006), participants performed an instrumental choice task, in which on each trial they had to choose one of two actions in order to either win money or avoid losing money. Activation in the medial OFC, a region that has been previously implicated in encoding stimulus reward value, was increased following the delivery of the reward, but also following successful avoidance of monetary loss.

From a clinical perspective, it seems noteworthy that although avoidance behavior prevents patients from encountering the feared outcome (e.g., pain), it—ironically—leads to heightened fear and catastrophic thinking in the long-term (Craske et al., 1989; Eifert and Heffner, 2003). In line with this notion, fear-related activation in the amygdala and insula seem to be maintained even when aversive outcome is avoided (Schlund et al., 2010), confirming that avoidance preserves rather than erases fear.

Taken together, studies on avoidance learning suggest that avoidance behavior might have a rewarding component that could explain its maintenance, even if it is associated with high costs—an aspect we will explore in the next section. It should be noted that in studies on avoidance learning, aversive outcome has so far commonly been operationalized as loss of monetary reward or absence of gains to allow for direct comparison of positive and negative outcome (i.e., gain vs. loss of money). Whether findings from these studies can directly be translated to the delivery of aversive stimuli such as pain and on a more general level to avoidance behavior related to acute and chronic pain warrants further investigation.

Although to date research on avoidance behavior has mainly focused on learning, related aspects could aid in understanding the motivational basis of this behavior and its common resistance to extinction. For instance, dispositional inter-individual differences in exploratory behavior that might be determined by personality or genotype could add a relevant piece to the puzzle of understanding and targeting excessive avoidance behavior. Furthermore, contemporary theories on action selection suggest that our behavior is governed by at least two systems, a goal-directed system and a habitual system (see Rangel et al., 2008 for review). Avoidance behavior might require different intervention strategies, depending on the system driving it. If the behavior is goal-directed (or “model-based,” see Daw and Shohamy, 2008 for details), it could be targeted by challenging its underlying beliefs—an approach that is, for instance, indicated when avoidance behavior is driven by exaggerated irrational beliefs. In contrast, if the behavior is habitual, it might subsist despite successful treatment of pain that caused the avoidance behavior.

### Goal conflict in the context of pain

Although avoidance behavior might help in reducing pain on the short-term, it is often associated with immediate and long-term costs. Giving up on the plan to watch a movie at the cinema might spare one the back pain from sitting in an uncomfortable chair but also deprives from the pleasure of spending time with friends. Moreover, conflict can also arise from approach behavior. For instance, because long-term consumption of certain analgesics is known to increase the risk of side effects, the momentary pain relief has to be compared against the health risk associated with consumption of the analgesic. The urge to avoid pain can therefore compete with other interests we have. Of note, the perception of goal conflict itself can be distressing and might even contribute to symptom exacerbation (Hardy et al., 2011).

Contemporary models of goal-directed choices (e.g., Rangel and Hare, 2010) posit that the decision whether to pursue an action (e.g., pursuing physical activity in the presence of pain) or not depends on the value of this action that results from the difference between the value of the outcome that is generated by each action (e.g., pleasure experienced during physical activity) and the associated costs (e.g., increase in pain).

There is now solid evidence from numerous studies in animals and humans showing that stimulus evaluation as the first part of this equation critically depends on a region comprising parts of the ventromedial prefrontal cortex (VMPFC) and orbitofrontal cortex (OFC; see Levy and Glimcher, 2012). Interestingly, the OFC seems to be concerned with the evaluation of appetitive stimuli as well as aversive stimuli (Plassmann et al., 2010; Morrison and Salzman, 2011).

Experiments exploring the relevance of costs commonly investigate changes in the evaluation of desired outcome (e.g., monetary reward) when it co-occurs with aversive outcome such as loss of money or delivery of noxious stimuli. In a study by Talmi et al. (2009), participants had to choose between monetary reward that was associated with a low or high probability to receive a mild or strong electric shock. Their results show that although the OFC still signaled the reward value of expected payment, activation in this region was attenuated the stronger the expected noxious stimulation, suggesting that the OFC integrates costs into stimulus evaluation. This integrative mechanism was recently studied in more detail using a computational modeling approach in which behavioral data (i.e., response times and choice behavior) were employed to inform the analysis of neuroimaging data (Park et al., 2011). As in the study by Talmi and colleagues, participants could accept or reject offers that consisted of a combination of different amounts of monetary reward and pain of different intensity levels. Neuroimaging data in combination with computational modeling confirmed that both outcomes are considered in an interactive (non-linear fashion) in the OFC but also in the subgenual anterior cingulate cortex (sACC) and dorsolateral prefrontal cortex (DLPFC), suggesting that these regions integrate information about costs (e.g., pain) into the evaluation of expected benefits (i.e., money).

Although the prospect of pain can trigger avoidance behavior and it is often tempting to even abandon previously valued activities because they might lead to pain, we are sometimes able to “stay on task” (Seminowicz and Davis, 2007a) or pursue potentially pain-related activities despite the pain. In these cases, the value of an activity seems to outweigh the gain of pain avoidance. This suggests that higher-level goals such as the long-term outcome of a decision can influence the decision-making process and might even be considered at the stage of action value calculation. First evidence for such a top–down influence on stimulus or action evaluation comes from a study in which participants had to choose between healthy and unhealthy food of varying palatability (Hare et al., 2009). As in previous studies, the evaluation of the food engaged the VMPFC/OFC. However, trials in which participants opted for the healthy food were also characterized by increased activation in the DLPFC—the key region for top–down cognitive control. Most importantly, the engagement of the VMPFC during the presentation of liked-but-unhealthy food was reduced as a function of DLPFC involvement during these trials, suggesting that value encoding in the VMPFC is sensitive to input from a brain region representing higher-order goals.

To summarize, there is cumulative evidence suggesting that the prospect of pain is integrated into the evaluation of appetitive stimuli and might thereby affect the net evaluation of these stimuli. The translation of this experimental research in healthy volunteers into patients suffering from chronic pain could provide novel, clinically highly relevant insights into pain-related choices and more specifically, the compromised ability to implement top–down processing in goal conflicts. A particularly promising focus is the characterization of impaired DLPFC functions, which comprise not only a top–down influence on stimulus and action evaluation but also executive functions such as “goal shielding” through biased attentional processing. Furthermore, future neuroimaging studies on pain-related goal conflicts should consider other conflict-relevant dimensions apart from valence. In contrast to experimental settings in which participants choose between simple stimuli that are delivered immediately, conflict in the context of (chronic) pain often arises from more complex scenarios in which the options are typically on different time scales (e.g., pain relief from analgesics as short-term benefit vs. side-effects as long-term adversity). Insights into the integration of action outcome with different time constants could help in understanding the preference for immediate pain relief despite the detrimental long-term costs. Finally, future studies on the resolution of goal conflicts in the context of pain should explore the integration of relevant information in the brain in more detail. The exchange and comparison of information regarding costs and benefits as well as the subsequent decision-making processes require dynamic brain circuitries rather than single brain regions. Tools focusing on dynamic parameters (e.g., analysis of functional connectivity) and computational models that inform brain imaging analysis based on behavioral data can therefore add valuable new insights.

### Interruptive function of pain: attentional processes

Although top–down influences can aid in protecting goals unrelated to pain, they have to allow for vital information to enter awareness in order to ensure survival. Because of its biological relevance, pain is often prioritized over concurrent activities and can therefore disrupt ongoing cognitive processes (see Eccleston and Crombez, 1999 for review). In experimental studies, this interruptive function of pain is reflected in compromised accuracy and speed in cognitive tasks (e.g., Stroop task, dot-probe, primary task paradigm) when the task is performed during concomitant noxious stimulation in comparison to a condition in which the task is performed without noxious stimulation (Crombez et al., 2012; Moore et al., 2012).

In order to understand the disruptive effect of pain, we have to consider the way the brain copes with simultaneous attention-demanding processes. Contemporary models of attention hold that our attentional capacity is limited (Lavie, 2005) and concomitant cognitive processes compete for attentional resources. Highly demanding or prioritized processes would thereby engage full capacity in relevant processing and leave no spare capacity to other processes.

A number of findings from neuroimaging studies support the notion that a competition for common resources accounts for the interruptive function of pain. First, some brain regions including the prefrontal cortex, primary and secondary somatosensory cortex, rostral ACC, anterior insula, and cerebellum can be sensitive to both operations (Wiech et al., 2005; Seminowicz and Davis, 2007a). Second, the effect of pain on concomitant cognitive processes is most prominent the higher the pain intensity and the more difficult the task. While mildly and moderately painful stimuli often have no or only minor effects (Seminowicz and Davis, 2007a), more severe pain that is more likely to attract attentional resources can increase error rates (Wiech et al., 2005). In line with this observation, Buhle and Wager (2010) showed that the degree to which pain compromises task performance is directly proportional to the perceived intensity of pain on a trial-by-trial basis. These findings suggest that the increased demand for attentional resources when the task is performed under pain can be compensated for until no more resources can be allocated; then the lack of resources becomes apparent as either compromised task performance or attenuated pain perception.

Attentional resources are allocated to perceptual processes based on the salience of the incoming information as well as the relevance of the information for prioritized goals (for review see Legrain et al., 2009). Stimulus salience that is defined as the ability of a stimulus to stand out relative to other stimuli (Yantis, 2008) is highest for novel, intense and potentially threatening stimuli and commonly triggers bottom–up mechanisms of attention selection. Bottom–up attentional processes have mainly been related to the anterior insula and MCC and the salience network described above. Importantly, the anterior insula as the central hub of the salience network is connected to the cognitive control network. This network consists of the DLPFC and the posterior parietal cortex (PPC) and governs cognitive functions such as attention allocation, working memory and decision-making (for review see Katsuki, 2012). Once a stimulus has been detected as salient, the anterior insula activates the cognitive control network (Sridharan et al., 2008) and thereby facilitates task-related information processing. In other words, the anterior insula ensures that salient stimuli such as painful stimuli will have preferential access to the brain's attentional and working memory resources (Menon and Uddin, 2010). Moreover, the anterior insula decreases activity in the “default mode network, DMN” (Sridharan et al., 2008) that comprises the VMPFC and posterior cingulate cortex (PCC) and shows decreased activation during sensory or cognitive processing. Although the relevance of DMN modulation for selective attention is less well understood, there is evidence showing that failure of this DMN regulation through the anterior insula leads to inefficient cognitive control (Bonnelle et al., 2012). In line with these findings, patients with CLBP (Loggia et al., 2012) and those with fibromyalgia (Napadow et al., 2010) show a heightened functional connectivity between the anterior insula and the DMN that decreased with successful pain treatment in fibromyalgia patients (Napadow et al., 2012). Fibromyalgia patients in whom pain often co-occurs with cognitive impairments also showed an increased functional connectivity between the anterior insula and the cognitive control network that exhibits increased engagement during attention-demanding operations, including pain. In healthy individuals, a increased attentional demand as, for instance, during task performance under pain, can be accommodated for by an increase in the engagement of the cognitive control network that ensures consistent performance despite the pain (Seminowicz and Davis, 2007b). Although speculative at the moment, it is conceivable that this ability is compromised by the overriding influence of the anterior insula that prioritizes the more threatening operation.

The allocation for attentional resources, however, not only depends on stimulus salience but also on internal goals that are implemented by top–down signals from the cognitive control network, predominantly in the DLPFC as described above. Through the allocation of attentional resources, this system ensures focused attention on goal-relevant stimuli while responses to distractors in the presence of relevant stimuli are suppressed.

Importantly, pain not only interferes with the performance of cognitive operations but can also hamper concomitant perceptual processes. Using fMRI, Bingel et al. (2007) investigated the influence of concomitant application of noxious stimuli on visual processing. In this study, laser stimuli of different intensities were applied during performance of a working memory task (1- or 2-back task). The noxious stimulation lead to longer response times, particularly when the more demanding 2-back task had to be performed during high-intensity stimulation. In a subsequent surprise recognition task, participants showed lower recognition rates for pictures that had previously been presented with high-intensity stimulation. At the neural level, this interruptive effect of pain on task performance was reflected in impaired visual processing, as indicated by reduced activation in the lateral occipital complex during high pain.

To summarize, the high biological relevance of pain is likely to trigger the salience network that ensures prioritized processing through connections with the cognitive control network governing attention allocation. Although directing attention to pain is critical in acute situations to prevent further harm, it can lead to severe cognitive disability in chronic pain. Additional studies are needed to understand under which circumstances we are able to “stay on task” and how cognitive control regions ensure that we can disengage from pain. Coordinating demands and available resources requires communication between brain regions, which is likely to be reflected in dynamic parameters of a flexible network of brain regions. A more detailed understanding of the factors that guide the allocation of attentional resources could shed light on the over-prioritization of pain-related processes that is characteristic for many chronic pain syndromes and often interferes with the pursuit of goals unrelated to pain (see Van Damme et al., 2010). Inter-individual differences in the ability to recruit the top–down control might explain the different effects pain can have on task performance (Braver et al., 2010), including compromised task performance in some and improved performance in others (Seminowicz et al., 2004; Tiemann et al., 2010).

## Influence of motivational states on the perception of pain

For centuries, the perception of pain had been conceptualized as a linear read-out of incoming nociceptive information: the more nociceptive information enters the sensory system, the stronger the pain. However, over the recent years numerous studies have demonstrated that pain is substantially influenced by cognitive-affective processes, including motivational factors such as “fear of pain” or the prospect of pain relief. The following section will mainly focus on the influence of fear as one of the most basic motivations but will also highlight recent advances on the influence of social factors as a new, emerging field of research. For a discussion of other, more complex cognitive processes on pain, we refer the reader to two review articles (Wiech et al., 2008; Wiech and Tracey, 2009).

### Fear and anxiety

Amongst the different motivational states, the influence of fear and anxiety on pain has probably most extensively been studied. Numerous behavioral studies have shown that fear generally leads to higher pain intensity ratings and reduced pain tolerance (see Wiech and Tracey, 2009 for review). Ploghaus et al. (2001) were the first to demonstrate that the increase in pain perception during an experimental manipulation of anxiety leads to amplified processing in pain-related brain regions, including the insula and cingulate cortex which can be considered “target” regions of the anxiety-related modulation of pain. Subsequent studies focusing on cognitive aspects of fear and anxiety such as expectation, anticipation, or catastrophizing extended this finding. The expectation of high-intensity pain resulted in increased activation in pain-related brain regions during stimulus receipt relative to low-intensity expectation, despite physically identical stimulation (Koyama et al., 2005). Moreover, stimulus-related brain responses can be predicted based on the level of activation during the preceding anticipation period (Fairhurst et al., 2007; Ploner et al., 2010). Although the experimental manipulation used in these studies differ, they are all aimed at varying the threat or interruptive value of pain.

So far, only a few studies have aimed at identifying brain regions that might be involved in mediating the effect (i.e., “sources” of modulation). During stimulus application, the expectation of a high-intensity stimulus is associated with increased activation of the (para)hippocampal regions (Ploghaus et al., 2001; Gondo et al., 2012) and individuals who are sensitive to anxiety-inducing cues show stronger hippocampal activation during stimulus anticipation and receipt than those who are less cue-sensitive (Ziv et al., 2009). More importantly, the (para)hippocampal formation seems to be related to anxiety produced changes in activity in pain-related brain regions (i.e., ACC and mid/posterior insula) during a more threatening condition (Ploghaus et al., 2001). Similarly, activation in the hippocampal formation (and ventral tegmental area of the brainstem) predicted insular activity during stimulus delivery (Fairhurst et al., 2007). Furthermore, the hippocampus might also be involved in nocebo effects (Kong et al., 2008; Bingel et al., 2011). When healthy volunteers were instructed that the withdrawal from the potent analgesic remifential could amplify pain perception, the reported increase in pain ratings scaled with increased activation in the left hippocampus (Bingel et al., 2011). Together, these findings suggest that the hippocampal formation may “tune” the sensitivity of brain regions involved in pain processing in a context-dependent manner. This notion is in accordance with the Gray-McNaughton theory on the hippocampal function in fear and anxiety (Gray and McNaugthon, 2000) that posits that the hippocampus amplifies neural representations of aversive events in order to bias the organism toward a behavior that is most adaptive to the worst possible outcome, as stated in Ploghaus et al. (2001).

A fear-related modulation of pain regions through a change in communication between brain regions has also been shown for the anterior insula (Wiech et al., 2010). As mentioned in the section on the interruptive function of pain, the anterior insula ensures that salient stimuli such as painful stimuli will have preferential access to mental resources. Together with the MCC, it is a key node of a network that predominantly responds to salient stimuli (Seeley et al., 2007; Franciotti et al., 2009; Taylor et al., 2009). Importantly, the directive influence of the anterior insula is sensitive to momentary perceptions of fear and anxiety. Contextual information about the threat value of an upcoming, potentially painful stimulation, for instance, engages the anterior insula which increases its functional connectivity with the MCC while participants are awaiting the stimulation (Wiech et al., 2010). Importantly, participants who subsequently showed a higher tendency to rate ambiguous stimuli as painful were characterized by a stronger activation in the MCC during stimulus receipt, indicating that the “tuning” of the MCC is perceptually relevant. In keeping with the notion of the anterior insula as a central hub for the amplification of pain through fear and anxiety the change in functional connectivity between the anterior insula and the periaqueductal grey (PAG) as a key region of the descending pain inhibitory network was found to depend on the trait anxiety of participants during an experiment examining how pre-stimulation brain activity predicts whether near threshold stimuli are perceived as painful or not (Ploner et al., 2010). The pivotal role of the anterior insula in the modulation of pain through fear and anxiety was also confirmed in a formal mediation analysis that identified the anterior insula (and other regions) as critical for cue-related effects on pain perception (Atlas et al., 2010). In sum, these studies indicate that the anterior insula connects to regions involved in pain processing (e.g., MCC) and modulation (e.g., PAG) in a flexible, context-dependent fashion.

In addition to hippocampal regions and anterior insula, studies in chronic pain populations emphasize the role of prefrontal areas in fear and anxiety-related modulation of pain, albeit with a considerable variation in prefrontal location. During the anticipation of pain as a cognitive element of fear and anxiety, patients with Irritable Bowel Syndrome (IBS) showed increased activation in the ventrolateral prefrontal cortex (VLPFC; Lee et al., 2012) while increased activation in the dorsolateral aspect of the prefrontal cortex (DLPFC) was found in fibromyalgia patients relative to healthy controls (Burgmer et al., 2011). The DLPFC is known to orchestrate cognitive processes such as selective attention, working memory or emotion regulation by connecting to brain regions that are relevant for these processes. The VLPFC, in contrast, has mainly been implicated in emotion regulation (Mitchell, 2011). In line with this notion, Jensen et al. (2012) recently showed that a reduction in anxiety through CBT correlated with an increase in VLPFC activation in fibromyalgia patients. In addition to functional changes, chronic pain patients also show fear and anxiety-related structural alterations in prefrontal areas. For instance, patients with Complex Regional Pain Syndrome (CRPS) exhibit increased white matter connectivity between the VMPFC and nucleus accumbens (NAc) that was related to heightened anxiety (Geha et al., 2008).

Although fear and anxiety generally increase the perception of pain, the opposite effect can be found when these emotions exceed a certain level. From a motivational perspective, this so-called stress-induced analgesia is of particular interest because it demonstrates that pain can also be subject to priority considerations similarly to cognitive processes that can be disrupted by pain, as discussed above. If the individual is faced with challenges that are biologically more relevant than pain (i.e., survival in an acutely threatening situation) pain is perceived as less intense. Stress-induced analgesia is predominantly mediated by opioidergic mechanisms, as also reflected by the engagement of brain regions known to be part of the opioid-dependent descending pain inhibitory system, such as the rostral ACC (Yilmaz et al., 2010), but it also involves non-opioidergic (e.g., endocannabinoid) processes (Hohmann et al., 2005).

Despite recent advances in this field, additional studies are needed to understand the complex interaction between fear/anxiety and pain processing in more detail. First, a growing number of observations on the role of the (para-)hippocampal formation in pain modulation has to be integrated into the vast body of literature on this structure in fear and anxiety in general. Furthermore, the significance of this structure for pain-related and fear-related disruption of cognitive operations as discussed in the section on the interruptive function of pain warrants further investigation. For instance, a recent study showed that the pain-related disruption of memory encoding was reflected in the hippocampus (Forkmann et al., 2013), suggesting that this structure is not only a mediator of pain modulation but might also be a target. Although the hippocampus is often considered a single functional entity, there is cumulating evidence suggesting a functional segregation into a dorsal part related to cognitive functions and a ventral part that is involved in emotional processing and stress (for an overview see Fanselow and Dong, 2010) which also show differential functional connectivity patterns under threat (Satpute et al., 2012). The investigation of the role of both sub-divisions in pain-related fear and anxiety could reveal a more detailed picture of the relevance of the hippocampus in the modulation of pain.

Second, although a wealth of animal studies has highlighted the relevance of brainstem structures such as the PAG and VTA in fear-related pain modulation, precise insights into their role in human pain models are relatively sparse. However, the repeatedly found involvement of these structures in studies on cognitive-affective aspects in healthy volunteers (Bantick et al., 2002; Tracey et al., 2002; Dunckley et al., 2005; Fairhurst et al., 2007; Ploner et al., 2010; Brodersen et al., 2012; Buhle et al., 2012), human models of central sensitization (Iannetti et al., 2005; Zambreanu et al., 2005; Lee et al., 2008; Wanigasekera et al., 2011) and chronic pain patients (Berman et al., 2008) points toward an equally critical role in humans. Second, studies outside the pain field have emphasized the significance of the amygdala and its dynamic interaction with prefrontal regions in fear and anxiety (Bishop, 2007). Although a recent study suggested a decrease in amygdala activity as a robust indicator for successful emotion and pain regulation (Lapate et al., 2012), our understanding of amygdala function in human pain processing is still limited to its role in associative learning, whereas for animal studies it has a well characterized role in nociceptive processing (Neugebauer et al., 2004; Ji et al., 2010). Future studies should therefore investigate the translation of these animal models into humans. Third, the variability in findings on prefrontal cortex contribution warrants further investigation. Studies on the role of the prefrontal cortex in cognitive control and emotion regulation have, for instance, inspired hierarchical models whereby the lateral prefrontal cortex controls anxiety-related limbic activity through connections with the VMPFC (Klumpers et al., 2010). Studies with a focus on prefrontal function, probably probing its involvement in pain modulation using transcranial magnetic stimulation (TMS) could detail the notion of “keeping pain out of mind” (Lorenz et al., 2003) as the key function of the prefrontal cortex in pain modulation.

### Placebo analgesia, reward and dopaminergic transmission

The type of pain modulation that has probably most commonly been linked to motivational aspects is placebo analgesia. More specifically, it has been hypothesized that the ability to produce an analgesic effect via endogenous pain inhibitory mechanisms scales with the anticipation of reward from pain relief (for a more comprehensive view on placebo analgesia, including the role of the descending pain inhibitory pathway in mediating the influence of placebo-related beliefs, see Zubieta and Stohler, 2009; Tracey, 2010; Atlas and Wager, 2012). Using functional molecular imaging, Scott et al. (2007) investigated the relationship between reward anticipation and individual analgesic placebo responses in healthy volunteers. Their results showed that the degree of placebo analgesia correlated with the release of dopamine during placebo analgesia. Moreover, both measures were proportional to activation in the NAc during the expectation of monetary reward in a separate fMRI experiment, which indicates that variations in the function of reward processing might determine one's ability for endogenous pain control.

But what exactly is the link between the dopaminergic system and (endogenous) analgesia? There is evidence suggesting that dopamine itself might have analgesia properties and might affect nociceptive processing directly (for an overview see Jarcho et al., 2012). Another possibility, however, that has been proposed in the context of placebo analgesia as a form of endogenous pain modulation and that is of particular interest from a motivational perspective is the notion that dopaminergic NAc signal might be involved in the “encoding of the incentive value of the placebo, possibly acting as a gate or permissive system for the formation of placebo effects” (Scott et al., 2007). The expectation of reward (e.g., pain relief) triggers the release of dopamine in the NAc as the key structure of the ventral striatum. Studies on placebo effects in patients with Parkinson disease have shown that this expectancy-related release of dopamine in the ventral striatum precedes the release of dopamine in the dorsal striatum which leads to the placebo effect in patients with Parkinson disease (de la Fuente-Fernández et al., 2002). Analogously, NAc dopamine release could drive the release of endogenous opioids, as recently proposed by Fuente-Fernández (de la Fuente-Fernández, 2009). Although experimental evidence for this pathway is still missing, placebo-induced dopaminergic NAc activity has been found to be positively correlated with the activation of the μ-opioid system in brain regions showing a placebo effect (Scott et al., 2008). Given the correlative nature of this finding, it is, however, difficult to discern whether the release of dopamine preceded or followed the release of opioids.

The relevance of the dopaminergic system for the modulation of pain has recently also been highlighted in a number of studies in chronic pain patients. Patients with fibromyalgia syndrome, for instance, showed reduced dopamine release following noxious stimulation in comparison to healthy controls (Wood et al., 2007). While the amount of dopamine release scaled with the perceived pain intensity in controls, such correspondence could not be found in the patient group. Furthermore, Geha et al. (2008) found substantial atrophy in the gray matter of the NAc in patients with CRPS patients. This finding is particularly interesting given that gray matter density in regions such as the ventral striatum (comprising the NAc) and prefrontal cortex is directly related to the degree of analgesia healthy volunteers experienced in a placebo paradigm (Schweinhardt et al., 2009). However, such changes are not consistent, as another study examining structural changes in patients with rheumatoid arthritis observed an increase in gray matter content in the basal ganglia, mainly in the NAc and caudate nucleus (Wartolowska et al., 2012). Finally, in a recent longitudinal study, Baliki et al. (2012) showed that the functional connectivity between NAc and prefrontal regions predicted the transition from acute to chronic back pain. Of note, it has recently been shown that mesolimbic dopaminergic regions including the NAc are controlled by the DLPFC (Ballard et al., 2011), linking reward processing and (placebo) analgesia to top–down control mechanisms that are involved in implementing higher-level goals.

Taken together, these studies suggest a critical role of dopaminergic reward-related brain regions and their interaction with the endogenous opioid system in pain modulation. However, direct evidence, for instance, from studies using dopamine antagonists in a placebo paradigm is still missing.

### Social influences

Although pain is a highly subjective and rather personal experience, it is sensitive to social influence. So far, the emerging strand of research on the influence of social factors on pain perception has mainly focused on two aspects: pain modulation through social support and social threat. Social support has been found to alleviate experimental and clinical pain, including labor, cardiac, and postoperative pain (see Brown, 2003 for an overview). In line with this change in pain intensity, participants exhibited less threat-related activation in various brain regions (including the anterior insula, DLPFC, and hypothalamus) when they were holding the hand of their spouse while they were awaiting a painful stimulation than when they were holding the hand of a stranger or in a non-hand-holding condition (Coan et al., 2006). Interestingly, this buffering effect was stronger the higher participants rated the quality of their marriage. In a recent study, Eisenberger et al. (2011) extended these observations to the period of pain receipt. Here, participants reported less pain when they were presented with a picture of their romantic partner during the application of the noxious stimuli. This modulatory effect was paralleled by increased activation in the VMPFC and as in the study by Coan et al. it scaled with perceived partner support. Moreover, activity in the VMPFC was related to decreased engagement of the dorsal anterior cingulate cortex (dACC) during pain receipt. Based on the association of the VMPFC with safety signaling (e.g., Klumpers et al., 2010) the authors concluded that social support might modulate pain via top–down regulatory mechanisms.

In comparison to this work on social support, the neural basis of the modulation of pain through social threat is less clear. Animal studies indicate that the relationship between social threat and pain perception might depend on the level of threat. In a study in mice, Langford and colleagues found reduced pain behavior in an experimental pain model when male animals were confined to close proximity to a stranger animal (Langford et al., 2011)—a finding that is in accordance with observations on stress-induced analgesia. However, when both animals were separated by metal bars that only allowed for partial physical contact and thereby reduced social stress, the same stimuli induced more pronounced pain behavior.

In addition to the level of threat, the effect of social threat also seems to depend on the perceived level of intentionality to cause harm. Physical harm that was caused by another person might be the result of an act of aggression or it might have occurred accidently. Interestingly, intentional harm is perceived as more severe and prevents habituation relative to non-intentional harm (Gray and Wegner, 2008; Peeters and Vlaeyen, 2011). Furthermore, the perceived intentionality seems to influence whether the (facial) expression of pain of the threatened individual corresponds to his perception of pain. Peeters and Vlaeyen (2011) showed that although intentional harm led to higher pain intensity ratings (relative to non-intentional pain) it reduced the facial expression of pain. The authors interpreted their finding within the framework of an evolutionary perspective on pain (Williams, 2002) that posits that the expression of pain also has a communicative function. In this view, the communication of pain aids in soliciting empathy and social support. However, it also discloses a level of vulnerability that might be exploited by less benevolent others to cause further harm. The suppression of pain expressions in the face of social threat might therefore be the more adaptive response if further intentional harm has to be feared.

Research on social influences on pain is still in its infancy but has already proven to add valuable insights into a more comprehensive view on pain [for an excellent overview on motivational and learning aspects of pain communication see Hadjistavropoulos et al. (2011)]. Future studies could aid in understanding the specific neurobiology underlying persistent pain states caused through interpersonal violence (e.g., from torture), which are known to be particularly resistant to treatment.

## Outlook

In this review, we have discussed two aspects that highlight the strong link between pain and motivations: the fact that pain motivates decisions and actions to prevent harm to the organism and the observation that pain, in turn, is also subject to motivations. Together, these findings encourage a functional perspective on pain that sees pain not only as a somatosensory experience but focuses on the various repercussions it has for cognitive, affective and social processes and considers its motivational aspects. The primary aim of most treatment approaches to chronic pain is the identification of pathological processes that cause or maintain the pain. Although this approach is successful in many cases, a large number of patients still suffer from pain that modern medicine has no sufficient relief or cure for. The observations discussed in this review show that research into the motivational aspects of pain is not only key to a better understanding of mechanisms that maintain or even cause pain, but because of their causal link to the development and maintenance of (chronic) pain they also offer promising ways to prevent and treat pain.

**Figure 1:**
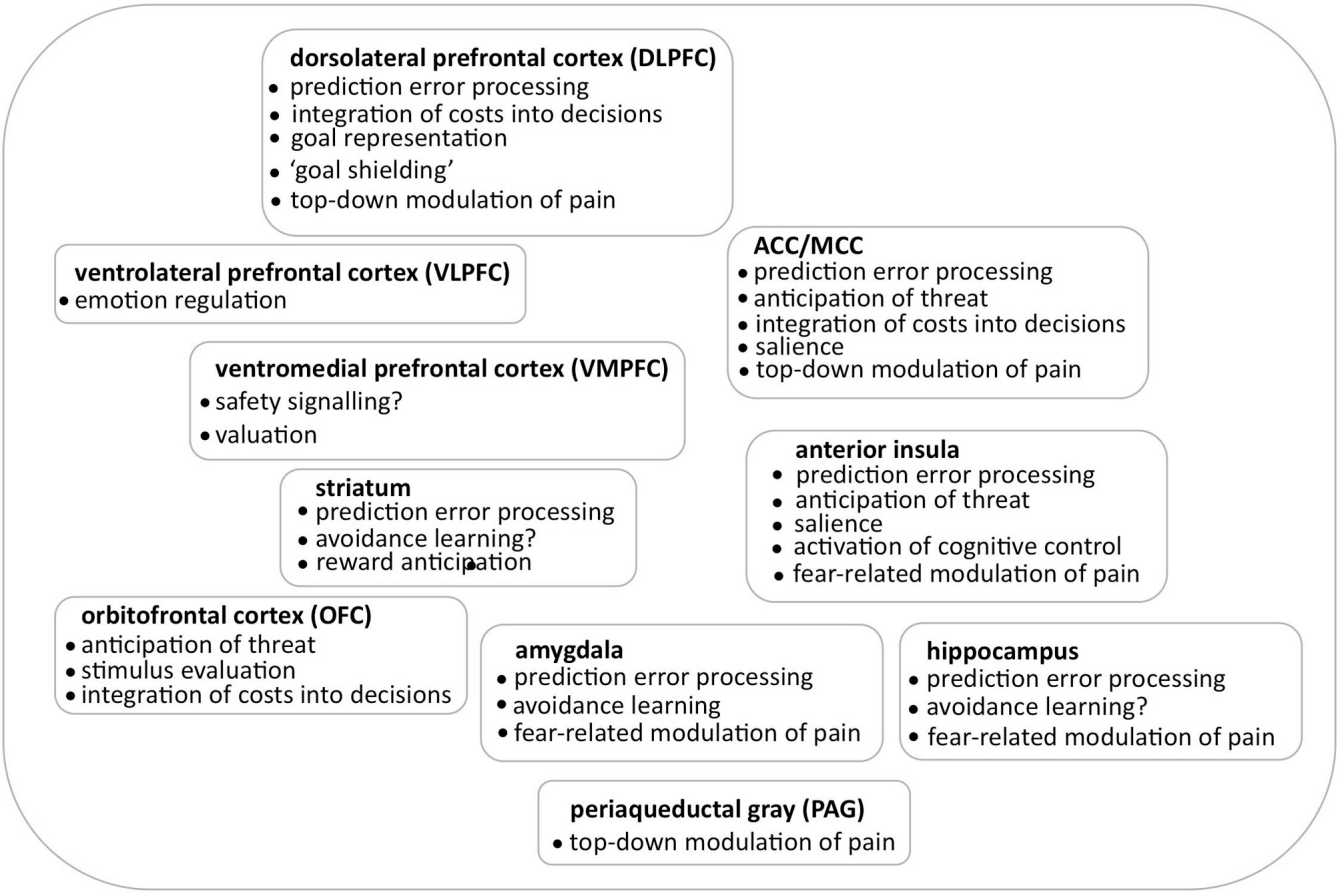
**Overview on the brain regions implicated in motivational aspects of pain**.

Over the recent years, considerable progress has been made in understanding motivational aspects of pain and identifying brain regions that are involved in these processes (for an overview see Figure 1). However, further research is needed to advance and refine these insights. First, studies need to go beyond the mapping of complex cognitive and psychological constructs to single brain areas and consider extended networks and their context-dependent dynamic reconfiguration. Advanced analysis techniques such as dynamic causal modeling allow for a detailed characterization of the cross-talk between brain regions, by specifying the direction of causation. Analyses of functional imaging data that are informed by results on the structural connectivity of relevant brain regions in the same individual will provide more insights into the individual capacity for pain modulation. Furthermore, recent advances in computational models can aid in characterizing relevant processes in more detail by using behavioral data such as response times to inform neuroimaging analyses.

Second, neuroimaging studies on motivational aspects of pain would benefit from the transfer and integration of findings on related topics, including fear and anxiety, decision-making, conflict resolution and goal-directed behavior. Research on anxiety, for instance, has shown that compromised prefrontal top–down processing underlies the attentional bias in high trait-anxious individuals (Bishop, 2009)—a mechanism that might also underlie biased attentional processing in chronic pain patients. Likewise, it has been shown that long-term consequences affect stimulus evaluation less than short-term consequences, a phenomenon termed temporal discounting. Similar processes might influence the decisions chronic pain patients make when comparing the immediate benefit of pain avoidance with the loss from missing out on previously valued activities.

Another aspect that has only received very little attention is the motor implications of pain. Pain undoubtedly motivates withdrawal behavior, particularly in acute situations, and drives behavior requiring motor responses in the chronic situation. Motor implications of pain are notoriously difficult to investigate using neuroimaging techniques, given the movement-related confounds they produce. However, understanding the (cognitive) demand of motor implications and their suppression could add a missing piece to the puzzle of pain.

Chronic pain remains one of the largest unresolved medical health problems in the developed world. A better understanding of how the brain responds in an adaptive and maladaptive way during the transition to and maintenance of chronic pain is key if we are to target these mechanisms for better patient management, pain relief and well being.

### Conflict of interest statement

The authors declare that the research was conducted in the absence of any commercial or financial relationships that could be construed as a potential conflict of interest.
